# Supplementary material for: Enhancement of Thermal Management Performance of Copper Foil Using Additive–Free Graphene Coating
Source: Polymers (Basel). 2024 Jun 30;16(13):1872. doi: 10.3390/polym16131872 (PMC11244482; doi:10.3390/polym16131872)
Supplement: Supplementary file 1 [file polymers-16-01872-s001.zip › polymers-3055225-supplementary.pdf]

Supplementary Material

Enhancement of thermal management performance of copper foil using additive-free graphene coating

Bing Hu, Huilin Yuan, Guohua Chen*

College Materials Science and Engineering Huaqiao University, 668 Jimei Blvd, Xiamen Fujian, 361000, China;

Binguuh@163.com (B.H.); yuanhlll@163.com (H.Y.)

* Correspondence: hdcgh@hqu.edu.cn; Tel.: +86-0592-662280

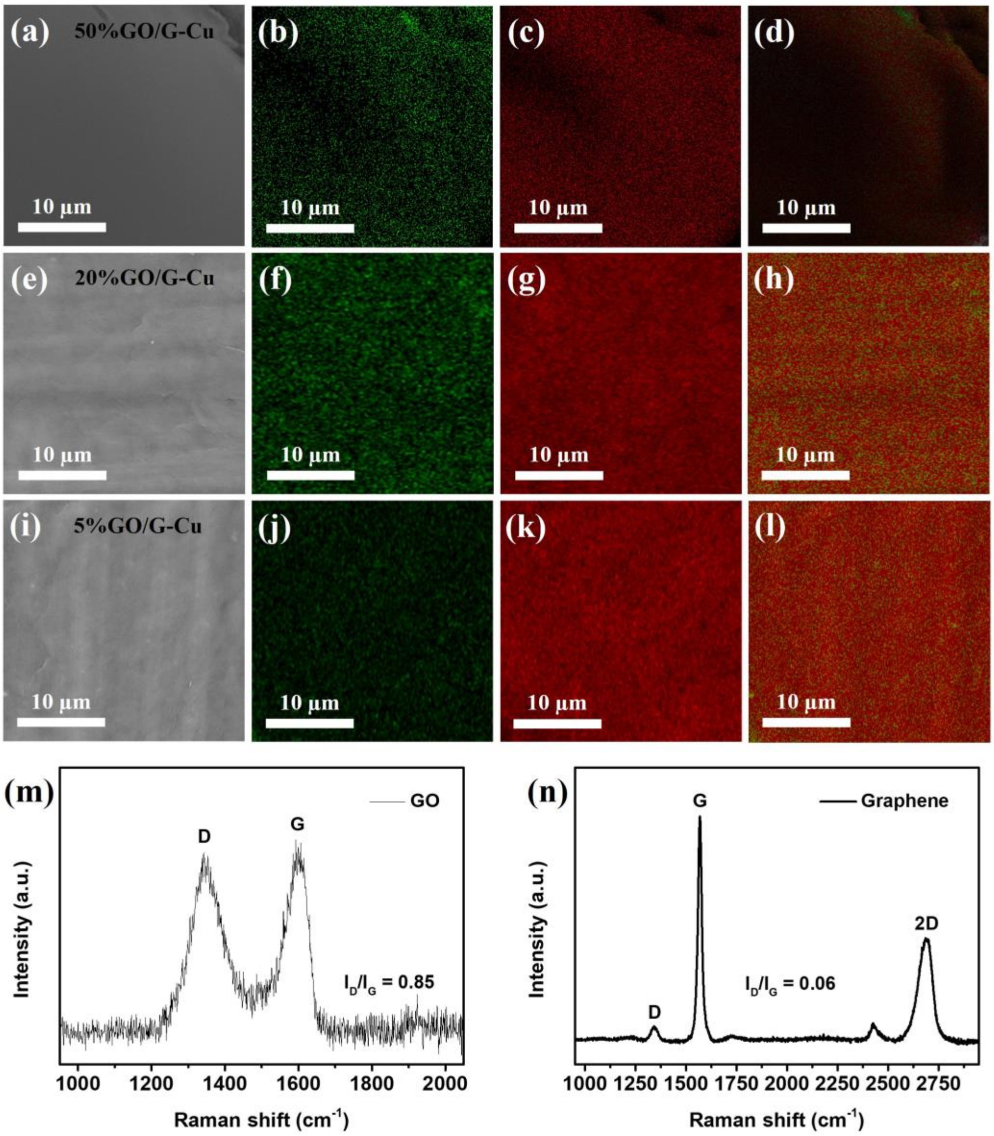

Figure S1. The SEM image (a, e, i) and the corresponding EDS mapping (b–d), (f–h), (i–l) of composite films, O (green) and C (red). Raman spectra of GO (m) and Graphene (n).

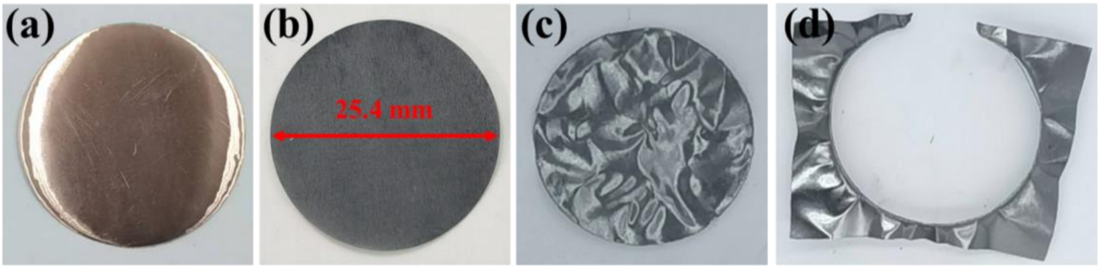

Figure S2. Copper foil surface (a); Coating layer (b); Flexible display diagram of the 10% rGO/G-Cu (c-d).

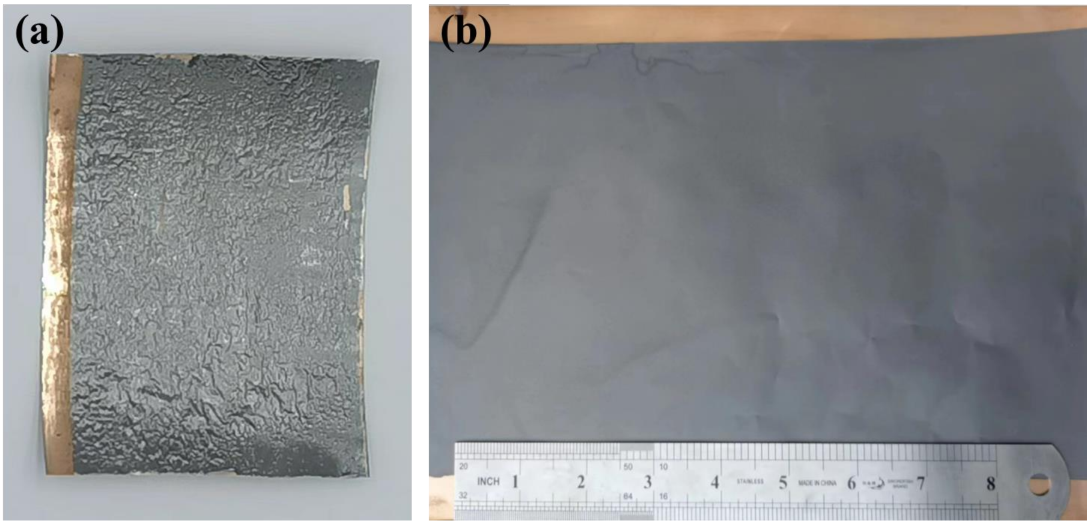

Figure S3. Pure graphene-copper foil composite film (a); 10% rGO/G-Cu composite film prepared in large size.

Table S1 Content of carbon and oxygen elements in EDS analysis of all samples.

Sample	C (wt%)	O (wt%)
50% rGO/G-Cu	80.68	19.32
20% rGO/G-Cu	89.84	10.16
10% rGO/G-Cu	90.80	9.20
5% rGO/G-Cu	93.02	6.98
10% PVA/G-Cu	91.76	8.24
